# Multilevel functional network signature of Pisa syndrome in Parkinson’s disease: a resting-state fMRI study

**DOI:** 10.3389/fnagi.2026.1862635

**Published:** 2026-07-16

**Authors:** Zhou Su, Mengran Liu, Jun Kuai, Congcong Wang, Bin Xu, Li Su, Min Su, Yuechang Zheng, Junyu Peng, Yinghao Yang, Tingting Yi

**Affiliations:** 1 Department of Neurology, The First Affiliated Hospital of Henan Medical University, Xinxiang, Henan, China; 2 Henan Medical Key Laboratory of Neurology, Henan Joint International Laboratory of Neurorestoratology for Senile Dementia, Henan Key Laboratory of Neurorestoratology and Protein Modification, Xinxiang, Henan, China; 3 School of Education, University of Bristol, Bristol, United Kingdom; 4 Department of Gastroenterology, The First Affiliated Hospital of Henan Medical University, Xinxiang, Henan, China; 5 Department of Laboratory Medicine, The Second Affiliated Hospital of Henan Medical University, Xinxiang, Henan, China; 6 Department of Radiology, The Second Affiliated Hospital of Henan Medical University, Xinxiang, Henan, China

**Keywords:** brain networks, functional connectivity, Parkinson’s disease, Pisa syndrome, resting-state fMRI

## Abstract

**Background:**

Pisa syndrome (PS) is a disabling postural complication of Parkinson’s disease (PD), but its functional network basis remains insufficiently defined. The aim of this study is to determine whether PD with PS is associated with multilevel resting-state functional network abnormalities beyond differences in motor severity.

**Methods:**

We studied 31 patients with PD and PS and 42 patients with PD without PS using resting-state fMRI. Analyses included seed-based functional connectivity, regional homogeneity, fractional amplitude of low-frequency fluctuations, and graph-theoretical metrics of whole-brain topology. Group comparisons adjusted for age, sex, education, Hoehn and Yahr (H&Y) stage, and levodopa equivalent daily dose (LEDD); sensitivity analyses further adjusted for the Movement Disorder Society-Unified Parkinson’s Disease Rating Scale Part III (MDS-UPDRS-III).

**Results:**

Compared with PD without PS, PD-PS showed reduced connectivity between the pedunculopontine nucleus (PPN) and the left supplementary motor area (SMA) and right cerebellar Crus I, reduced interoccipital connectivity, increased connectivity between the right anterior insula and posterior cingulate/medial prefrontal cortex, lower occipital and postcentral fALFF, lower right superior parietal ReHo, and disrupted whole-brain topology with lower small-worldness and efficiency and longer path length. PPN-SMA connectivity correlated inversely with MDS-UPDRS-III and positively with visuospatial/executive performance, but not with lateral flexion angle; global efficiency correlated with Hoehn and Yahr stage and lateral flexion angle. PPN-related findings reflect connectivity involving a PPN-centered brainstem region rather than the PPN nucleus per se. Findings remained significant after additional adjustment for MDS-UPDRS-III.

**Conclusion:**

PS in PD is associated with convergent abnormalities involving brainstem-sensorimotor coupling, visual-parietal integration, salience-default mode interactions, and global network organization. These findings demonstrate group differences in functional network organization between PD patients with and without PS. They support a multilevel network model, but further studies are needed to determine whether these alterations are specifically related to PS severity or pathophysiology.

## Introduction

Pisa syndrome (PS) is a disabling postural deformity characterized by lateral trunk flexion (Doherty et al., 2011). It was first used to describe a secondary trunk muscle tone disorder after treatment with antipsychotic drugs (Ekbom et al., 1972). Subsequently, PS has been recognized as a clinically relevant complication of Parkinson’s disease (PD) (Castrioto et al., 2014), secondary parkinsonism (Marsili et al., 2019), and other neurodegenerative disorders (Sławek et al., 2006; Noda et al., 2015; Woo et al., 2020; Su et al., 2022).

Current evidence suggests that dysfunction of the nigrostriatal pathway, impaired sensory information processing, cognitive deficits, and altered somatosensory orientation perception contribute to the pathophysiology of PS (Tinazzi et al., 2015; Barone et al., 2016). However, the pathophysiology of PS remains debated. Some studies have emphasized peripheral mechanisms, including axial muscle imbalance, paraspinal muscle asymmetry, and secondary musculoskeletal changes (Doherty et al., 2011; Tinazzi et al., 2015). Others have pointed to central nervous system dysfunction, particularly involving the pedunculopontine nucleus (PPN), basal ganglia-thalamocortical circuits, and higher-order sensory integration areas (Ricciardi et al., 2014; Huh et al., 2018). Clinical and neuroimaging studies point toward a network-level dysfunction that disrupts the brain’s intricate capacity for sensorimotor integration, leading to an impaired perception of verticality and a distorted body schema (Huh et al., 2018; Biassoni et al., 2023). Some studies have demonstrated that PD patients with PS exhibit marked deficits in verticality perception and visuospatial function, suggesting a distorted internal representation of the body’s orientation in space (Artusi et al., 2023a; Artusi et al., 2023b; Jiang et al., 2025). Furthermore, clinical associations, such as Rapid Eye Movement Sleep Behavior Disorder (RBD) as a predictor of PS, suggest a shared neurodegenerative substrate, potentially centered on the pedunculopontine nucleus (Ricciardi et al., 2014; Liu et al., 2019; Sasaki et al., 2022).

Resting-state functional magnetic resonance imaging (rs-fMRI) provides a powerful tool to investigate the brain’s functional architecture (Cieri et al., 2024). In PD, alterations in the default mode network (DMN), sensorimotor network, and visual networks have been linked to motor and cognitive symptoms (Baik et al., 2024; Shi et al., 2025). We acknowledge that peripheral factors may contribute to PS; however, the aim of this study was not to adjudicate between central and peripheral mechanisms but to test the specific hypothesis that PS is associated with a detectable and reproducible pattern of resting-state functional network alterations. Decreased functional connectivity between the insula and the supplementary motor area (SMA) has been linked to gait dysfunction in PD patients with postural abnormalities (Yao et al., 2020). Furthermore, research has revealed that dynamic functional connectivity alterations and a progressive “network collapse” are central to cognitive decline (Brown et al., 2025). These findings provide a compelling theoretical framework for investigating PS, suggesting it may represent a distinct “network disorder” within the PD (Yao et al., 2020). However, a systematic characterization of the functional brain network alterations underlying PS in PD remains in its infancy.

Despite this growing evidence, a systematic, multilevel characterization of functional brain network alterations in PD patients with PS remains lacking. Most prior studies have focused on a single type of connectivity measure (e.g., seed-based functional connectivity) or a single regional metric, leaving it unclear whether PS is associated with abnormalities at only one level of brain organization or across multiple levels (local synchrony, regional activity intensity, circuit-level connectivity, and global topology) (Piramide et al., 2024; Liao et al., 2026). We hypothesized that PD patients with PS would exhibit a hierarchically organized pattern of network alteration characterized by (1) reduced PPN-seeded connectivity with cortical and cerebellar sensorimotor regions; (2) dysfunction within visual and multisensory integration systems corresponding to the clinically observed visuospatial deficits; and (3) reduced global efficiency and small-world organization of whole-brain networks. To test this multilevel hypothesis, we employed three complementary analytical approaches: seed-based functional connectivity, regional homogeneity (ReHo), fractional amplitude of low-frequency fluctuations (fALFF), and graph-theoretical analysis. Accordingly, this study aimed to characterize functional network alterations associated with PS in PD rather than to infer causality or diagnostic specificity.

## Materials and methods

### Study participants

This cross-sectional study included 73 patients with PD, who were recruited from the outpatient and inpatient wards of the Department of Neurology between December 2021 and September 2025. They were divided into two groups: 31 PD patients with Pisa syndrome (PD-PS group) and 42 PD patients without Pisa syndrome (PD-nPS group). The two groups were matched for age, sex, and years of education. PD patients were diagnosed according to the Movement Disorder Society Clinical Diagnostic Criteria for Parkinson’s disease (Postuma et al., 2015). Patients meeting the following exclusion criteria were excluded from this study: (1) Presence of severe neurological or psychiatric conditions, including severe visual/auditory impairment, aphasia, limb paralysis, or severe mental disorders, and/or inability to complete required clinical evaluations due to the aforementioned conditions; (2) Diagnosis of atypical parkinsonism (e.g., multiple system atrophy, progressive supranuclear palsy, and dementia with Lewy bodies) or secondary parkinsonism.

Patients with PS exhibited trunk lateral flexion of at least 10°, as measured by a wall-mounted goniometer (Doherty et al., 2011). The lateral flexion angle was measured with the patient standing in a relaxed, natural posture, feet shoulder-width apart, and arms hanging freely. A vertical reference line was established using the goniometer’s gravity-dependent scale. The examiner (a trained neurologist with >5 years of experience in movement disorders) then measured the coronal angle between the vertical line and a line passing through the spinous processes of the C7 and L4 vertebrae. The measurement was performed twice on each patient, and the average value was recorded. Patients with postural disorders (e.g., primary dystonia, scoliosis >10°, antecollis ≥45°), major spinal surgery, musculoskeletal lesions (e.g., myopathy, myositis, ankylosing spondylitis), or those unable to complete postural assessment or who had received treatment with medications potentially inducing postural instability within 6 months prior to enrollment were excluded. This study was approved by the Medical Ethics Committee of the First Affiliated Hospital of Henan Medical University (EC-024-523). Informed consent was obtained from participants or their legal guardians in accordance with the ethical principles of the Helsinki Declaration.

### Clinical data collection and neuropsychological assessment

This present study collected demographic data, medical history, clinical symptoms, and medication history. The levodopa equivalent daily dose (LEDD) was calculated for each patient. All patients with PD were scanned in the practical ON state, defined as having taken their regular morning dose of antiparkinsonian medication approximately 1–2 h before the start of the MRI session. This standardized timing was followed for all participants to minimize within-group variability. A comprehensive neuropsychological assessment was carried out by well-trained neuropsychologists face-to-face. Global cognition was evaluated using the Montreal Cognitive Assessment (MoCA) (Smith et al., 2007). Depressive symptoms were measured using the Hamilton Depression Rating Scale (HAMD) (Hamilton, 1967). Motor symptoms were assessed using the Movement Disorder Society-Unified Parkinson’s Disease Rating Scale Part III (MDS-UPDRS-III) (Goetz et al., 2008) and the Hoehn and Yahr (2011) (H&Y) stage. Daily living functioning was evaluated employing the Activities of Daily Living (ADL) Scale (Bouça-Machado et al., 2022). Formal diagnosis of Parkinson’s disease mild cognitive impairment (PD-MCI) was not assigned because the present study was not designed as a diagnostic cognitive cohort.

### Image data acquisition

All participants underwent rs-fMRI and high-resolution T1-weighted structural imaging on a Siemens 3.0 T MRI platform using the same acquisition parameters for the analyzed datasets. The rs-fMRI data were acquired using an echo-planar imaging (EPI) sequence with the following parameters: repetition time (TR) = 2000 ms, echo time (TE) = 30 ms, flip angle = 90°, field of view (FOV) = 240 × 240 mm^2^, matrix size = 64 × 64, slice thickness = 4 mm, no inter-slice gap, and 240 volumes. The total acquisition time for the resting-state scan was 8 min (240 volumes × 2.0 s TR, after discarding the first 10 volumes for signal stabilization, the remaining 230 volumes corresponded to 7 min and 40 s of usable data). High-resolution T1-weighted images were obtained with a 3D magnetization-prepared rapid gradient-echo sequence: TR = 1900 ms, TE = 2.52 ms, 176 sagittal slices, and isotropic voxel size = 1 mm^3^. Participants were instructed to remain awake with eyes closed during scanning. Immediately after each scanning run, participants completed a brief post-scan questionnaire that asked, “(1) Did you feel drowsy during the scan?” (yes/no), and (2) “If yes, for approximately what proportion of the scan did you feel drowsy?” (<25%, 25–50, >50%). Participants who answered “yes” and reported drowsiness for >25% of the scan were considered to have significant drowsiness. Data were reacquired if motion artifacts or significant drowsiness were detected.

### Data preprocessing

Functional imaging data were preprocessed using the Data Processing & Analysis for Brain Imaging (DPABI V5.1) toolkit^1^ running on MATLAB R2018a (MathWorks, Natick, MA, USA) (Yan et al., 2016). The first 10 volumes were discarded to eliminate transient signal instability during scanner equilibrium. Slice timing correction and rigid-body head-motion realignment were then performed. The functional images were co-registered to individual T1-weighted structural images and spatially normalized to the Montreal Neurological Institute 152 template using nonlinear transformation (resampled to 3 mm^3^ isotropic voxels) (Fonov et al., 2011). After nuisance regression of 24 head motion parameters (Friston model), cerebrospinal fluid, white matter signals, and the global mean signal, linear detrending was applied. To assess the potential impact of global signal regression (GSR) on our key seed-based functional connectivity findings (right anterior insula, PPN, and visual network seeds), we performed a sensitivity analysis in which the global signal was not regressed out (i.e., without GSR), while keeping all other preprocessing steps identical. Thereafter, modality-specific processing branches were used. For seed-based functional connectivity and ReHo analyses, images were spatially smoothed with a Gaussian kernel (full width at half maximum [FWHM] = 6 mm) and band-pass filtered (0.01–0.08 Hz) (Weissenbacher et al., 2009; Yan et al., 2016). For fALFF, calculations were performed on detrended, nuisance-regressed data without prior temporal band-pass filtering, because fALFF quantifies the proportion of low-frequency power relative to the full measurable spectrum (Friston et al., 1996). We retained GSR to reduce widespread non-neuronal variance and motion-related contamination, while interpreting functional connectivity findings cautiously in view of the known influence of this step on correlation structure (Murphy and Fox, 2017). To control for motion artifacts, framewise displacement (FD) was calculated for each volume. Participant-level exclusion was applied: any participant whose rs-fMRI dataset contained more than 20% of volumes with FD > 0.2 mm was excluded from all subsequent analyses. For the remaining participants, mean FD (averaged across all volumes) was calculated to quantify residual head motion at the participant level for group comparison.

### Functional connectivity and network analysis

The analytical strategy was hypothesis-driven and designed to interrogate complementary levels of brain organization relevant to PS, rather than to perform an unrestricted search across imaging metrics (Zhou, 2019). Firstly, seed-based functional connectivity analysis was performed to probe connectivity patterns from key regions of interest implicated in PS pathophysiology. Seed regions of interests (ROIs) were selected based on prior pathophysiological and network literature and included (1) PPN, a key cholinergic hub for posture and gait; (2) the subthalamic nucleus (STN), involved in sensorimotor and nociceptive integration; (3) the left occipital cortex, representing the visual network; and (4) the right anterior insula, representing the salience network. The PPN and STN seeds were defined as a 5 mm radius spheres centered at MNI coordinates (±9, −25, −16) for PPN, (±12, −12, −8) for STN, (−18, −96, −6) for left occipital cortex, and (36, 22, −8) for right anterior insula, based on previously published coordinates derived from a histologically validated brainstem atlas and used in prior PD rs-fMRI studies (Gallea et al., 2017; Yao et al., 2020; Joza et al., 2022; Artusi et al., 2023b). Spherical ROIs were generated using the DPABI toolkit. For each seed, the mean time series was extracted and correlated with the time series of every other voxel in the brain using Pearson’s correlation. The resulting correlation maps were then Fisher’s r-to-z transformed to normalize the distribution for group-level statistical analysis.

We employed three complementary analytical approaches, each targeting a distinct level of brain functional organization. ReHo quantifies the local synchronization of spontaneous neural activity by calculating Kendall’s coefficient of concordance between the time series of a given voxel and its 26 nearest neighbors. In the context of PS, abnormal local synchronization within sensory-motor and parietal regions could reflect disrupted micro-circuitry underlying the integration of somatosensory and spatial information. fALFF measures the relative contribution of low-frequency oscillations (0.01–0.08 Hz) to the total power spectrum, thereby indexing the intensity of regional spontaneous neural activity without requiring *a priori* seed selection. PS related postural abnormalities may involve not only connectivity changes but also primary alterations in the “excitability” of occipital and somatosensory cortices, which provide afferent inputs for postural control. While ReHo and fALFF capture local or regional properties, graph theory quantifies the global topological organization of whole-brain functional networks. Given that axial posture requires rapid, parallel integration of multisensory information across distributed brain regions, PS may be associated not only with focal abnormalities but also with a breakdown of efficient small-world architecture, such as global efficiency, local efficiency, and characteristic path length, providing a systems-level characterization of network disruption.

To investigate the global topological organization of brain networks, we performed graph theoretical analysis using GRETNA v2.0.0^2^ (Wang et al., 2015). Whole-brain functional networks were constructed by parcellating the brain into 116 ROIs according to the Automated Anatomical Labeling 3 atlas (Rolls et al., 2015). For each subject, a 116 × 116 functional connectivity matrix was generated by computing pairwise Pearson correlations between the mean time series of all ROIs. A consistent sparsity-based threshold (range: 0.10–0.30, step = 0.01) was applied across subjects to retain a fixed number of edges for comparable network density (Rubinov and Sporns, 2010). Key global topological metrics were then calculated, including small-worldness (*σ*), global efficiency (Eglob), local efficiency (Eloc), and characteristic path length (Lp) (Liao et al., 2017). For each subject, we computed the four global topological metrics (σ, Eglob, Eloc, Lp) at each of the 21 sparsity thresholds. We then calculated the area under the curve (AUC) for each metric across the entire sparsity range. AUC integration is the recommended approach in network neuroscience because it avoids arbitrary threshold selection and does not require multiple comparison correction across thresholds (Rubinov and Sporns, 2010; Liao et al., 2017).

### Statistical analyses

All statistical analyses and data management were performed using SPSS 26.0 for Mac. Continuous variables were presented as mean ± standard deviation (SD) when normally distributed or as median (interquartile range) for non-normally distributed data. Between-group comparisons were performed using Student’s *t*-test for parametric data and the Mann–Whitney U test for nonparametric data. Categorical variables were summarized as frequencies (*n*) with percentages and analyzed using the chi-square test or Fisher’s exact test. For neuroimaging data, group comparisons of functional connectivity, ReHo, and fALFF maps were conducted using two-sample t-tests within DPABI, with age, sex, education, H&Y stage, and LEDD included as covariates of no interest. Multiple comparisons for voxelwise analyses (seed-based functional connectivity, ReHo, and fALFF) were corrected using Gaussian Random Field (GRF) theory with a combined threshold of voxel-level *p* < 0.001 and cluster-level *p* < 0.05. However, cluster-wise inference is known to have limitations; Eklund et al. (2016) demonstrated that certain cluster-based thresholds can yield inflated false-positive rates under some conditions. We therefore employed a relatively stringent voxel-level threshold (*p* < 0.001). Group differences in graph theory metrics were assessed using analysis of covariance (ANCOVA) applied to the AUC values of each metric (small-worldness, global efficiency, local efficiency, characteristic path length), controlling for the same set of covariates. Because AUC integration summarizes performance across 21 thresholds into a single value per metric per subject, no additional correction for multiple threshold comparisons was necessary. Effect sizes were calculated as partial eta squared (ηp^2^), with values of 0.01, 0.06, and 0.14 interpreted as small, medium, and large effects, respectively. Spearman’s correlation analysis was employed to explore the relationships between significantly altered neuroimaging metrics (e.g., PPN connectivity strength, visual network integrity, fALFF/ReHo in key clusters) and clinical measures (e.g., PS lateral flexion angle, MDS-UPDRS-III, and MoCA visuospatial/executive). To further address the potential confounding effect of motor symptom severity, we performed a sensitivity analysis by re-running the voxelwise and graph-theoretical analyses with the MDS-UPDRS-III added and the HAMD score as a covariate. For the clinical-imaging correlations, Benjamini-Hochberg false discovery rate (FDR) correction was additionally applied. Using G Power 3.1 with *α* = 0.05 (two-tailed) and the actual sample sizes (*n*_1_ = 31, n_2_ = 42), the achieved power was 0.94 for global efficiency (partial η^2^ = 0.183, *f* = 0.47) and 0.89 for PPN-SMA connectivity (Cohen’s d = 0.85). These *post hoc* estimates should be interpreted cautiously, as they are based on observed effect sizes. All *p*-values reported are two-tailed, and *p* < 0.05 was considered statistically significant.

## Results

Based on the demographic and clinical characteristics presented in Table 1, patients with PD and PS showed no significant differences compared to those without PS in terms of age, sex distribution, years of education, and disease duration (all *p* > 0.05). Similarly, no significant difference was observed in LEDD between the two groups (*p* = 0.334). Patients with PS exhibited significantly greater disease severity as measured by the H&Y stage (*p* < 0.001) and higher scores on the MDS-UPDRS-III (28.5 ± 11.2 vs. 17.3 ± 10.5, *p* < 0.001). ADL scores were significantly worse in the PS group (45.2 ± 12.6 vs. 32.8 ± 9.4, *p* < 0.001). Additionally, depressive symptoms were more severe in PD patients with PS (14.6 ± 8.5 vs. 10.1 ± 7.2, *p* = 0.012). Regarding cognitive function, while the total MoCA score did not differ significantly between groups, patients with PS performed worse on the visuospatial/executive (3.2 ± 1.4 vs. 3.9 ± 1.3, *p* = 0.021) and attention (4.8 ± 1.2 vs. 5.4 ± 0.9, *p* = 0.012) subdomains. No significant differences were observed in other cognitive domains.

**Table 1:** Demographic and clinical features of PD with and without PS.

Characteristics	PD with PS (*n* = 31)	PD without PS (*n* = 42)	Statistical test	*p value*
Age, years	68.2 ± 6.5	66.8 ± 7.1	*t* = 0.92	0.361
Male, *n* (%)	25 (64.1)	26 (61.9)	*χ*^2^ = 0.04	0.835
Education, years	9.5 ± 3.8	10.1 ± 3.5	*t* = −0.73	0.469
Disease duration, years	7.8 ± 4.2	6.9 ± 3.8	*t* = 1.02	0.310
Lateral Flexion Angle, °	15.3 ± 4.7	—	—	—
LEDD, mg/day	545.6 ± 352.1	475.3 ± 295.4	*t* = 0.97	0.334
H&Y stage	2.5 (2.0–3.0)	1.5 (1.5–2.0)	U = 485.5	<0.001
MDS-UPDRS-III	28.5 ± 11.2	17.3 ± 10.5	*t* = 5.98	<0.001
ADL score	45.2 ± 12.6	32.8 ± 9.4	*t* = 5.08	<0.001
HAMD score	14.6 ± 8.5	10.1 ± 7.2	*t* = 2.58	0.012
MoCA total score	22.5 ± 4.1	23.8 ± 3.7	*t* = −1.51	0.135
Visuospatial/executive	3.2 ± 1.4	3.9 ± 1.3	*t* = −2.35	0.021
Naming	2.6 ± 0.8	2.7 ± 0.7	*t* = −0.61	0.545
Attention	4.8 ± 1.2	5.4 ± 0.9	*t* = −2.58	0.012
Language	1.8 ± 0.9	2.1 ± 0.8	*t* = −1.59	0.116
Abstraction	1.2 ± 0.7	1.3 ± 0.6	*t* = −0.71	0.478
Memory	2.3 ± 1.5	2.8 ± 1.6	*t* = −1.45	0.151
Orientation	5.6 ± 0.8	5.7 ± 0.6	*t* = −0.65	0.519
Mean FD, mm	0.12 ± 0.05	0.11 ± 0.04	*t* = 0.96	0.341

PD, Parkinson’s disease; PS, Pisa syndrome; LEDD, Levodopa Equivalent Daily Dose; H&Y, Hoehn and Yahr; MDS-UPDRS-III, Movement Disorder Society-Unified Parkinson’s Disease Rating Scale Part III; ADL, Activities of Daily Living; HAMD, Hamilton Depression Scale; MoCA, Montreal Cognitive Assessment; FD, Framewise displacement. *p* < 0.05 significant difference.

Participant flow and motion quality control for rs-fMRI were shown in Table 2. Initially, 36 PD-PS and 48 PD-nPS participants underwent rs-fMRI. Among these, 3 PD-PS and 4 PD-nPS scans were reacquired due to motion artifacts, and 2 PD-PS and 3 PD-nPS scans were reacquired due to significant drowsiness (>25% of scan duration) reported on the post-scan questionnaire. After reacquisition, 4 PD-PS and 6 PD-nPS participants were excluded at the participant level because their fMRI datasets contained more than 20% of volumes with framewise displacement (FD) > 0.2 mm. One additional PD-PS participant was excluded due to an incidental structural abnormality on T1-weighted imaging. The final imaging sample consisted of 31 PD-PS and 42 PD-nPS participants. Head motion quality control revealed no significant between-group difference in mean FD for the final sample (PD-PS: 0.12 ± 0.05 mm; PD-nPS: 0.11 ± 0.04 mm; t = 0.96, *p* = 0.341).

**Table 2:** Participant flow and motion quality control for rs-fMRI.

Item	PD with PS	PD without PS	Statistical test	*p* value / Notes
Initial participants scanned	36	48	—	Participants who completed the initial rs-fMRI scan
Scans reacquired due to motion artifacts	3	4	—	Same participants rescanned immediately; no participant excluded at this stage
Scans reacquired due to drowsiness (post-scan questionnaire)	2	3	—	Participants reporting drowsiness >25% of scan duration; rescanned on same day
Participants excluded after reacquisition (excessive motion >20% volumes FD > 0.2 mm)	4	6	—	Excluded at participant level; did not enter final analysis
Other exclusions (technical failure/incomplete data)	1	0	—	One PD-PS participant excluded due to incidental structural abnormality
Final imaging sample included in analyses	31	42	—	Derived from the final analyzed cohort
Mean FD (mm) of final sample	0.12 ± 0.05	0.11 ± 0.04	*t* = 0.96	0.341

PD, Parkinson’s disease; PS, Pisa syndrome; FD, Framewise displacement.

The graph theory analysis of the whole-brain functional network, as presented in Table 3, indicates that the global topological architecture of PD patients with PS is significantly disrupted. Compared to the non-PS group, the PS group exhibited significantly lower small-worldness (*σ*; 1.31 ± 0.14 vs. 1.49 ± 0.11; *p* < 0.001), global efficiency (0.18 ± 0.03 vs. 0.23 ± 0.04, *p* < 0.001), and local efficiency (0.33 ± 0.05 vs. 0.39 ± 0.06, *p* = 0.002), along with increased characteristic path length (2.81 ± 0.39 vs. 2.41 ± 0.35, *p* < 0.001), after controlling for age, sex, education, H&Y stage, and LEDD. Sensitivity analysis with additional adjustment for MDS-UPDRS-III scores confirmed the robustness of the primary findings. In the graph theory analysis, all global topological differences between groups remained statistically significant after further controlling for motor severity: small-worldness (*F* = 8.52, *p* = 0.005), global efficiency (*F* = 10.67, *p* = 0.002), local efficiency (*F* = 6.98, *p* = 0.010), and characteristic path length (*F* = 9.23, *p* = 0.003). In contrast to the PPN and network seeds, the STN seed revealed no significant group differences in functional connectivity between PD-PS and PD-nPS after GRF correction (voxel-level *p* < 0.001, cluster-level *p* < 0.05). Exploratory analysis at a lower threshold (voxel-level *p* < 0.01, uncorrected) also did not reveal any consistent pattern.

**Table 3:** Global graph theory metrics of whole-brain functional networks.

Graph metric	PD with PS (*n* = 31)	PD without PS (*n* = 42)	Direction (PD-PS vs. PD-nPS)	Original *F*-value (ANCOVA)	Original *p*-value	Original ηp^2^	Sensitivity F-value (ANCOVA + MDS-UPDRS-III)	Sensitivity *p*-value	Sensitivity ηp^2^
Small-worldness (σ)	1.31 ± 0.14	1.49 ± 0.11	PD-PS < PD-nPS	12.87	<0.001	0.155	8.52	0.005	0.108
Global efficiency	0.18 ± 0.03	0.23 ± 0.04	PD-PS < PD-nPS	15.43	<0.001	0.183	10.67	0.002	0.135
Local efficiency	0.33 ± 0.05	0.39 ± 0.06	PD-PS < PD-nPS	10.91	0.002	0.135	6.98	0.010	0.091
Characteristic path length (Lp)	2.81 ± 0.39	2.41 ± 0.35	PD-PS > PD-nPS	13.56	<0.001	0.162	9.23	0.003	0.118

PD, Parkinson’s disease; PS, Pisa syndrome; MDS-UPDRS-III, Movement Disorder Society-Unified Parkinson’s Disease Rating Scale Part III. Data are presented as mean ± standard deviation. ANCOVA, analysis of covariance, controlling for age, sex, education, H&Y stage, and LEDD. ηp^2^ = partial eta squared (effect size). Sensitivity analysis additionally controlled for MDS-UPDRS-III. *p*-values indicate statistical significance (*p* < 0.05).

**Figure 1:**
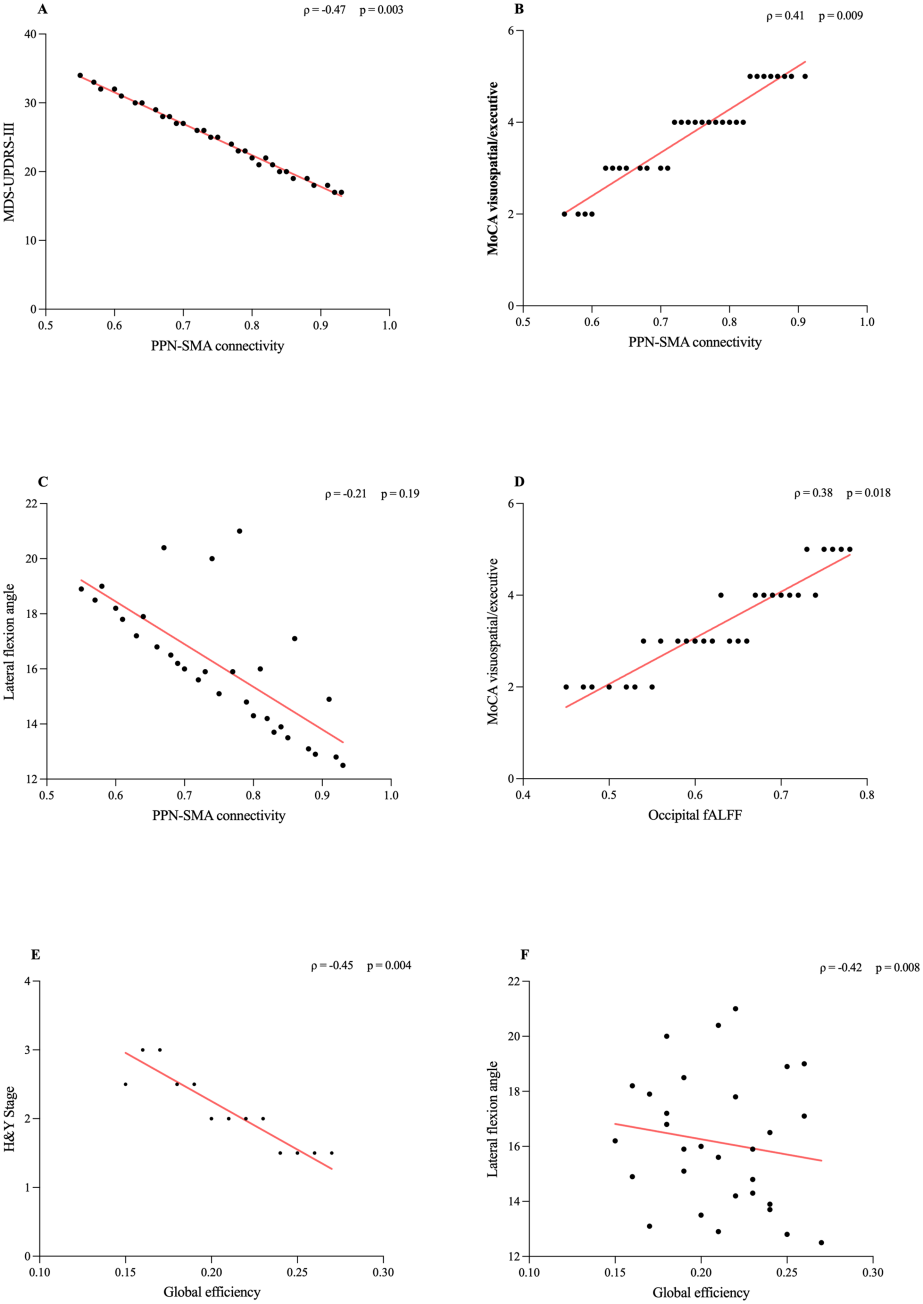
Scatterplots of clinical‑imaging associations in the PD‑PS group. Each panel displays individual data points (filled circles) with a linear trend line for visualization. Spearman’s rank correlation coefficient (ρ) and two‑tailed *p*‑value are shown in each panel. **(A)** PPN‑SMA connectivity versus MDS‑UPDRS‑III. **(B)** PPN‑SMA connectivity versus MoCA visuospatial/executive. **(C)** PPN‑SMA connectivity versus lateral flexion angle (non‑significant). **(D)** Occipital fALFF versus MoCA visuospatial/executive. **(E)** Global efficiency versus H&Y stage. **(F)** Global efficiency versus lateral flexion angle.

Table 4 indicates a significant correlation between functional network alterations in PD patients with PS and clinical disease severity. PPN-SMA connectivity was negatively correlated with MDS-UPDRS-III scores (*ρ* = −0.47, *p* = 0.003) and positively correlated with MoCA visuospatial/executive scores (*ρ* = 0.41, *p* = 0.009). Occipital fALFF was positively correlated with visuospatial/executive performance (*ρ* = 0.38, *p* = 0.018). The association between PPN-SMA connectivity and lateral flexion angle was not significant (Spearman’s *ρ* = −0.21, *p* = 0.19). Moreover, decreased global efficiency correlated with higher H&Y stage (*ρ* = −0.45, *p* = 0.004) and greater lateral flexion angle (*ρ* = −0.42, *p* = 0.008). All five associations remained significant after FDR correction (*q* ≤ 0.018). *Post-hoc* Spearman correlation analyses between LEDD and each of the key imaging metrics are shown in Table 5. No significant correlations were found between LEDD and any of these imaging metrics in the combined cohort (all *ρ* < 0.20, all *p* > 0.20). Scatterplots of the associations listed in Table 4 are provided in Figure 1.

**Table 4:** Associations between functional network alterations and clinical measures of disease severity in PD patients with PS.

Imaging Metric	Direction in PD-PS vs. PD-nPS	Clinical Measure	Spearman’s *ρ*	*p*-value
PPN-SMA connectivity	PD-PS < PD-nPS	MDS-UPDRS-III	−0.47	0.003
PPN-SMA connectivity	PD-PS < PD-nPS	MoCA visuospatial/executive	0.41	0.009
PPN-SMA connectivity	(PD-PS < PD-nPS)	Lateral flexion angle	−0.21	0.19
Occipital fALFF	PD-PS < PD-nPS	MoCA visuospatial/executive	0.38	0.018
Global efficiency	PD-PS < PD-nPS	H&Y Stage	−0.45	0.004
Global efficiency	PD-PS < PD-nPS	Lateral flexion angle	−0.42	0.008

PPN, Pedunculopontine nucleus; SMA, Supplementary motor area; MDS-UPDRS-III, Movement Disorder Society-Unified Parkinson’s Disease Rating Scale Part III; MoCA, Montreal Cognitive Assessment; fALFF, Fractional amplitude of low-frequency fluctuations; H&Y, Hoehn and Yahr. All correlations were calculated using Spearman’s rank correlation coefficient. *p* < 0.05 significant difference.

**Table 5:** Associations between LEDD and key imaging metrics.

Imaging metric	Spearman’s ρ	*p*-value
PPN-SMA connectivity	−0.12	0.31
Occipital fALFF (left middle occipital gyrus)	0.08	0.51
ReHo (right superior parietal lobule)	−0.10	0.40
Global efficiency	−0.15	0.21
Local efficiency	−0.11	0.36
Small-worldness (σ)	−0.09	0.45
Characteristic path length (Lp)	0.13	0.28

LEDD, Levodopa Equivalent Daily Dose; PPN, Pedunculopontine nucleus; SMA, Supplementary motor area; fALFF, Fractional amplitude of low-frequency fluctuations; ReHo, Regional Homogeneity. All correlations were calculated using Spearman’s rank correlation coefficient. *p* < 0.05 significant difference.

**Figure 2:**
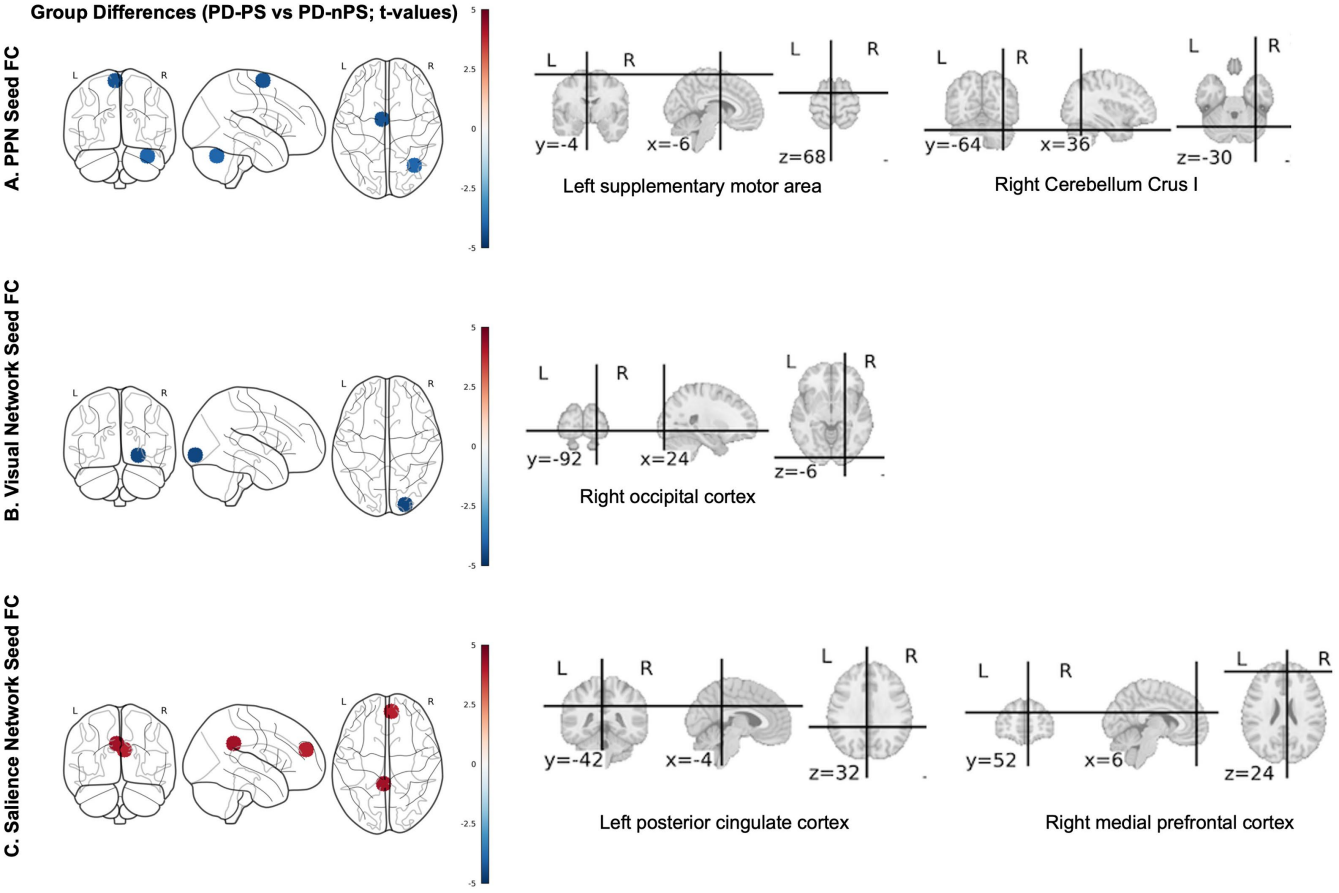
Group differences in seed-based functional connectivity between PD patients with Pisa syndrome (PD-PS) and those without (PD-nPS). **(A)** PPN seed: decreased connectivity (cool colors, negative t-values, PD-PS < PD-nPS) with left supplementary motor area (peak MNI: −6, −4, 68; T = −4.32; GRF-corrected cluster-level *p* = 0.001) and right cerebellum Crus I (peak MNI: 36, −64, −30; T = −3.95; GRF-corrected cluster-level *p* = 0.004). **(B)** Visual network seed (left occipital cortex): decreased connectivity (PD-PS < PD-nPS) with right occipital cortex (peak MNI: 24, −92, −6; T = −4.56; GRF-corrected cluster-level *p* < 0.001). **(C)** Salience network seed (right anterior insula): increased connectivity (warm colors, positive t-values, PD-PS > PD-nPS) with left posterior cingulate cortex (peak MNI: −4, −42, 32; T = 4.21; GRF-corrected cluster-level *p* = 0.001) and right medial prefrontal cortex (peak MNI: 6, 52, 24; T = 3.88; GRF-corrected cluster-level *p* = 0.003).

As shown in Figure 2 and Table 6, whole-brain seed-based functional connectivity analysis revealed significant alterations in PD patients with PS compared to those without PS. Decreased connectivity (PD-PS < PD-nPS) was observed between the PPN and the left SMA (peak MNI: −6, −4, 68; T = −4.32; GRF-corrected cluster-level *p* = 0.001) and the right cerebellum (Crus I; peak MNI: 36, −64, −30; T = −3.95; GRF-corrected cluster-level *p* = 0.004). Given the 3 mm isotropic resolution of our functional images and the small size of the PPN (≈10 mm), these findings should be interpreted as altered connectivity involving a PPN-centered brainstem region rather than definitive evidence of isolated PPN nucleus dysfunction. Additionally, the visual network seed in the left occipital cortex showed reduced connectivity (PD-PS < PD-nPS) with the right occipital cortex [Brodmann area 18 (BA18) 18; peak MNI: 24, −92, −6; T = −4.56; GRF-corrected cluster-level *p* < 0.001]. In contrast, the salience network seed in the right anterior insula exhibited increased connectivity (PD-PS > PD-nPS) with the left posterior cingulate cortex (peak MNI: −4, −42, 32; T = 4.21; GRF-corrected cluster-level p = 0.001) and the right medial prefrontal cortex (peak MNI: 6, 52, 24; T = 3.88; GRF-corrected cluster-level *p* = 0.003).

**Table 6:** Sensitivity analysis of voxelwise findings after adding MDS-UPDRS-III and HAMD.

Analysis/Region	Direction (PD-PS vs. PD-nPS)	Peak MNI	Original statistic	Original GRF-corrected cluster-level *P*	Adjusted statistic (+ MDS-UPDRS-III)	Adjusted GRF-corrected cluster-level *P*	Adjusted T (+ HAMD)	Adjusted P (+ HAMD)	Without GSR T	Without GSR *P*
PPN seed – left SMA	PD-PS < PD-nPS	(−6, −4, 68)	T = −4.32	0.001	T = −3.51	0.012	—	—		
PPN seed – right cerebellum (Crus I)	PD-PS < PD-nPS	(36, −64, −30)	T = −3.95	0.004	T = −3.18	0.028	—	—		
Left occipital seed – right occipital cortex (BA18)	PD-PS < PD-nPS	(24, −92, −6)	T = −4.56	<0.001	T = −3.89	0.006	—	—		
Right anterior insula seed – left posterior cingulate cortex	PD-PS > PD-nPS	(−4, −42, 32)	T = 4.21	0.001	T = 3.45	0.014	3.89	0.008	3.62	0.21
Right anterior insula seed – right medial prefrontal cortex	PD-PS > PD-nPS	(6, 52, 24)	T = 3.88	0.003	T = 3.12	0.032	3.51	0.018	3.21	0.038
fALFF – left middle occipital gyrus	PD-PS < PD-nPS	(−30, −88, 10)	T = −4.12	0.002	T = −3.43	0.015	—	—		
fALFF – right calcarine cortex	PD-PS < PD-nPS	(18, −78, 8)	T = −3.97	0.003	T = −3.29	0.022	—	—		
fALFF – left postcentral gyrus	PD-PS < PD-nPS	(−42, −28, 52)	T = −3.76	0.008	T = −3.05	0.041	—	—		
ReHo – right superior parietal lobule	PD-PS < PD-nPS	(30, −52, 62)	T = −4.01	0.004	T = −3.22	0.026	—	—		

PPN, Pedunculopontine nucleus; SMA, Supplementary motor area; fALFF, Fractional amplitude of low-frequency fluctuations; MDS-UPDRS-III, Movement Disorder Society-Unified Parkinson’s Disease Rating Scale Part III; HAMD, Hamilton Depression Scale; MoCA, Montreal Cognitive Assessment; ReHo, Regional Homogeneity; GSR, global signal regression. All corrected *p*-values are cluster-level *p*-values obtained from Gaussian Random Field (GRF) theory with voxel-level *p* < 0.001 and cluster-level *p* < 0.05 (two-sided). The “Original GRF-corrected cluster-level P” column refers to the primary analysis controlling for age, sex, education, H&Y stage, and LEDD; the “Adjusted GRF-corrected cluster-level P” column additionally controls for MDS-UPDRS-III.

As shown in Table 6, after adding HAMD score as an additional covariate, the increased connectivity between the right anterior insula and the left posterior cingulate cortex (peak MNI: −4, −42, 32; T = 3.89; GRF-corrected cluster-level *p* = 0.008) and between the right anterior insula and the right medial prefrontal cortex (peak MNI: 6, 52, 24; T = 3.51; *p* = 0.018) remained significant. The increased insula-DMN connectivity remained significant: insula-left PCC (T = 3.62, GRF-corrected cluster-level *p* = 0.021) and insula-right mPFC (T = 3.21, *p* = 0.038), although T values were lower than with GSR (original T = 4.21 and 3.88, respectively).

**Figure 3:**
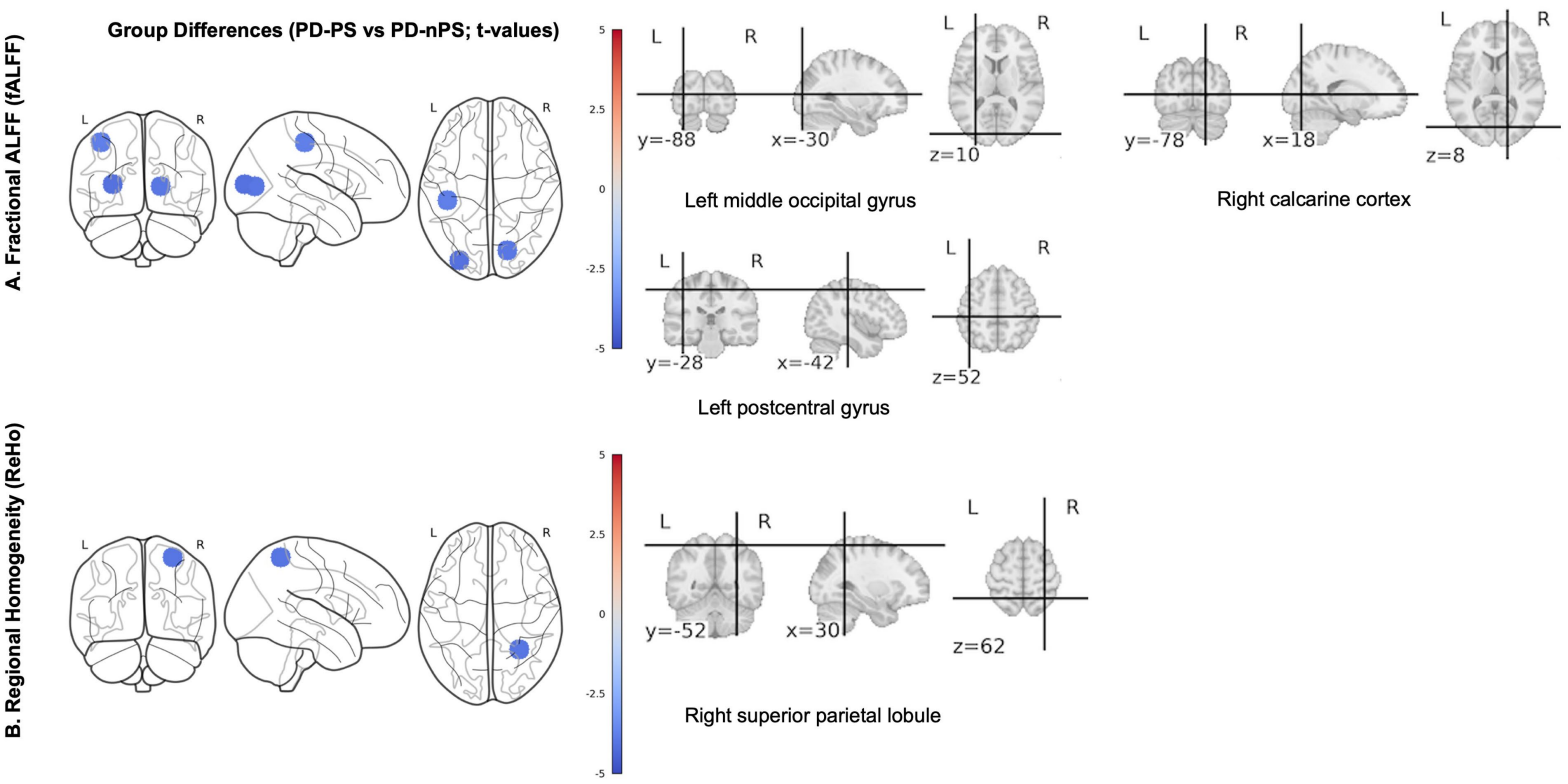
Group differences in regional brain activity between PD patients with Pisa syndrome (PD-PS) and those without (PD-nPS). **(A)** Fractional amplitude of low-frequency fluctuations (fALFF): decreased fALFF (cool colors, negative t-values; PD-PS < PD-nPS) in left middle occipital gyrus (peak MNI: −30, −88, 10; T = −4.12; GRF-corrected cluster-level *p* = 0.002), right calcarine cortex (peak MNI: 18, −78, 8; T = −3.97; GRF-corrected cluster-level *p* = 0.003), and left postcentral gyrus (peak MNI: −42, −28, 52; T = −3.76; GRF-corrected cluster-level *p* = 0.008). **(B)** Regional homogeneity (ReHo): decreased ReHo (cool colors, negative t-values; PD-PS < PD-nPS) in right superior parietal lobule (peak MNI: 30, −52, 62; T = −4.01; GRF-corrected cluster-level *p* = 0.004). Maps are thresholded at Gaussian Random Field (GRF) correction. Color bar represents *t*-values (range −5 to +5) from two-sample *t*-tests (PD-PS vs. PD-nPS). L, left; R, right.

As shown in Figure 3 and Table 6, analysis of regional neural activity demonstrated significant reductions in fALFF (PD-PS < PD-nPS) in PD-PS patients relative to PD-nPS in the left middle occipital gyrus (peak MNI: −30, −88, 10; T = −4.12; GRF-corrected cluster-level *p* = 0.002), right calcarine cortex (peak MNI: 18, −78, 8; T = −3.97; GRF-corrected cluster-level p = 0.003), and left postcentral gyrus (peak MNI: −42, −28, 52; T = −3.76; GRF-corrected cluster-level p = 0.008). ReHo was also significantly lower (PD-PS < PD-nPS) in the right superior parietal lobule (peak MNI: 30, −52, 62; T = −4.01; GRF-corrected cluster-level p = 0.004) in the PS group. The voxelwise group differences in seed-based functional connectivity, fALFF, and ReHo were all retained after adding MDS-UPDRS-III as an additional covariate, with corrected *p*-values remaining below 0.05.

## Discussion

This rs-fMRI study reveals multiple levels of functional network differences between PD patients with PS and those without PS. Our findings delineate a convergent pattern of neural abnormalities spanning reduced circuit-level functional connectibity, regional disturbances in spontaneous activity and local synchrony, and disruption of global network topology. Patients with PS group showed reduced functional connectivity between the PPN and key sensorimotor regions, reduced connectivity within the visual network, lower fALFF in occipital and postcentral cortices, lower ReHo in the superior parietal lobule, and a less efficient whole-brain architecture characterized by lower small-worldness, global efficiency, and local efficiency together with increased characteristic path length.

The presence of PS was associated with altered connectivity of the PPN with cortical sensorimotor systems. The PPN is widely regarded as a key cholinergic hub in the mesencephalic locomotor region, integral to posture, gait, and REM-sleep regulation (Gallea et al., 2017; He et al., 2021). The previously reported clinical association between RBD and later development PS has suggested a shared neural substrate involving the PPN (Liu et al., 2019). In our cohort, PD-PS patients showed weaker PPN connectivity with the SMA and cerebellum, two regions that are central to anticipatory postural adjustment, motor set formation, and online error correction of axial posture. The reduced PPN-SMA functional connectivity may reduce the efficiency with which postural information is transformed into adaptive motor commands. Whereas reduced PPN-cerebellar coupling may impair real-time recalibration of trunk position based on vestibular, proprioceptive, and efference-copy signals (Bohnen et al., 2014; Gallea et al., 2021). This confluence of disruptions situates the PPN’s weaker functional coupling with supra- and infratentorial centers, which may underpin the co-occurrence of sleep and postural disorders in PD (Bohnen et al., 2014; French and Muthusamy, 2018). Recent study emphasized that decreased functional connectivity among the insula, SMA, and middle frontal gyrus is a hallmark of PD with postural abnormalities, underscoring the vulnerability of this extended network (Li et al., 2024). This interpretation is consistent with the broader notion that postural abnormalities in PD emerge when brainstem locomotor and cortical motor systems fail to operate as an integrated control network (Chambers et al., 2019; Joza et al., 2022). However, the significant correlation of PPN-SMA connectivity with MDS-UPDRS-III (but not with lateral flexion angle) suggests that this finding may reflect general motor severity rather than a PS-specific mechanism. Although sensitivity analysis adjusted for UPDRS-III retained group differences, we caution against overinterpreting this connectivity alteration as uniquely associated with PS.

Our study identified group differences in visual and multisensory integration systems, which are thought to underlie disturbed verticality perception and altered body schema in PS. Reduced connectivity within the visual network, together with reduced fALFF in the occipital cortex, indicates diminished low-frequency neural activity in both primary and associative visual regions (Wang et al., 2023). Given the critical role of visual input in maintaining postural stability in PD, such functional impairments may directly contribute to distorted perception of verticality and spatial orientation (Kawabata et al., 2020). Additionally, decreased ReHo in the superior parietal lobule suggests localized disruption of neural synchrony (Jiang et al., 2016; Hu et al., 2019). Our study revealed that the PD-PS group demonstrated reduced connectivity between bilateral occipital regions, lower fALFF in the left middle occipital gyrus and right calcarine cortex, lower ReHo in the right superior parietal lobule, and lower fALFF in the left postcentral gyrus. These regions collectively may support the transformation of visual, somatosensory, and spatial information into an internal representation of body orientation. Moreover, the positive correlation observed between occipital fALFF values and performance on visuospatial tasks in our cohort reinforces the clinical significance of these sensory network deficits (Garcia-Diaz et al., 2018a; Garcia-Diaz et al., 2018b).

Clinical studies have shown that PS is frequently accompanied by impaired verticality perception and visuospatial dysfunction (Artusi et al., 2023a; Artusi et al., 2023b; Jiang et al., 2025), and our neuropsychological data similarly showed worse visuospatial/executive and attention performance in the PS group despite no significant between-group difference in total MoCA score. The positive correlation between occipital fALFF and visuospatial/executive performance further strengthens the clinical relevance of the imaging findings. From a pathophysiological perspective, reduced occipital and parietal network integrity could weaken the weighting of external spatial cues, whereas altered postcentral activity may compromise the somatosensory updating needed to keep the trunk aligned with perceived vertical. Such a combination would be expected to promote a persistent mismatch between perceived and actual body orientation, which is a plausible mechanism for lateral trunk deviation.

An additional finding was the increased connectivity between the right anterior insula and two canonical DMN regions, the posterior cingulate cortex and medial prefrontal cortex. This may reflect abnormal interactions between salience-detection systems and internally oriented self-referential networks. The anterior insula is thought to participate in switching between internal and external modes of processing; therefore, heightened insula-default mode coupling in PD-PS may represent either maladaptive network crosstalk or an attempted compensatory response to unreliable sensorimotor and visual inputs. This interpretation is also consistent with prior work linking abnormal insula-SMA connectivity to gait dysfunction in PD patients with postural abnormalities (Yao et al., 2020). In the setting of PS, altered salience-default mode interactions may contribute to inefficient allocation of attention during postural control, potentially helping to explain why attention performance was lower in the PS group.

The graph-theoretical findings indicate that PS is associated with an attenuated small-world organization (both groups still have *σ* > 1, but the PD-PS group shows a significantly reduced small-worldness). Lower small-worldness, reduced global efficiency, and longer characteristic path length indicate a less optimal balance between segregation and integration of information processing, whereas reduced local efficiency suggests lower fault tolerance of local subnetworks. Axial posture requires rapid integration of multisensory input with distributed motor output, a process that depends on both specialized local processing and efficient long-range communication. The correlation between lower global efficiency and both H&Y stage and lateral flexion angle indicates that global network disorganization tracks clinical severity within the PS group. These observations align with the broader concept of a progressive “network collapse” in neurodegenerative disease (Brown et al., 2025). Rather than viewing PS only as a consequence of severe motor disease, our results suggest that it may emerge when regional dysfunction in brainstem-sensorimotor and visual-parietal systems becomes embedded within a less efficient whole-brain network architecture.

Given that the higher H&Y stage and MDS-UPDRS-III scores in patients with PD and PS in our study analysis may affect the credibility of the results, we performed quality control and included related motor metrics in the analysis, after which the main voxel-level and graph-theoretic findings remained statistically significant. This suggests that the PPN-centered connectivity abnormalities, visuoparietal alterations, and impaired graph-theoretic metrics were not eliminated by further adjustment for motor severity. These fingdings indicate that the main results are not readily explained by motor severity alone and support that PS is associated with a distinctive pattern of network disruption within PD.

These findings across analytical levels may reflect a hierarchical cascade. At the local level, reduced occipital fALFF and parietal ReHo suggest weaker spontaneous activity and local synchrony in visual-somatosensory regions. At the circuit level, weaker PPN-SMA/cerebellar and inter-occipital connectivity indicate impaired sensorimotor and visual integration, while insula-DMN hyperconnectivity might represent compensatory recruitment of attentional networks. At the global level, reduced small-worldness and efficiency point to less efficient whole-brain information transfer. We propose that in PS, local sensory inefficiencies and brainstem-cerebellar coupling deficits place excessive demands on higher-order networks while global topology degrades, together forming a multilevel network signature.

This study has several limitations. First, the study was cross-sectional; the observed network alterations should be interpreted as associations rather than as demonstrated mechanisms, temporal sequences, or predictive biomarkers. Second, although we observed significant group differences in visuospatial/executive and attention performance, and these cognitive measures correlated with imaging metrics, we did not perform formal PD-MCI classification. Therefore, we cannot rule out the possibility that a higher rate of undiagnosed mild cognitive impairment in the PD-PS group, rather than PS itself, partially contributed to the cognitive differences and the imaging-cognition associations. Third, residual confounding by overall disease severity remains possible and limits the degree to which the current findings can be considered PS-specific. The sample size was modest and did not permit stage-stratified matching or more complex subgroup analyses. Although post-hoc power estimates for the primary group comparisons exceeded 0.85, these estimates are based on observed effect sizes and do not guarantee adequate power for detecting smaller effects or for interaction analyses. Fourth, standard rs-fMRI spatial resolution and spatial normalization do not permit definitive nucleus-specific inference for very small brainstem structures such as the PPN; accordingly, our results should be read as evidence of altered connectivity involving a PPN-centered brainstem region rather than as definitive evidence of isolated PPN dysfunction. Fifth, preprocessing decisions (such as global signal regression and ReHo/fALFF) may influence effect estimates. Sixth, even though we corrected for HAMD scores in sensitivity analyses, residual confounding from unmeasured features of mood disturbance or from the fact that HAMD is a clinical rating scale rather than a dimensional measure of depression severity cannot be totally eliminated. Seventh, we applied GSR in our primary preprocessing pipeline. GSR can introduce anticorrelation artifacts and may artificially inflate between-group differences. Nonetheless, we did not re-run fALFF, ReHo, or graph theory without GSR due to computational constraints; therefore, the possibility that results are influenced by GSR cannot be fully excluded. Eighth, the only measure of PS severity in this study was the lateral flexion angle measured by a wall-mounted goniometer. Therefore, the relationship between the observed functional network alterations and the severity of the postural deformity may be incompletely characterized. Finally, although all patients were scanned in the practical ON state, we did not perform a formal ON vs. OFF comparison. Moreover, we did not standardize the exact time post-dose or measure plasma levodopa levels. However, additional correlation analyses showed that LEDD did not correlate significantly with any of the key imaging metrics, reducing the likelihood that between-group differences in medication dosage accounted for the observed network alterations. Nonetheless, the acute effects of dopaminergic medication on rs-fMRI connectivity cannot be fully excluded, and further research is needed to clarify.

## Conclusion

PD patients with PS exhibited group differences compared to those without PS, including reduced PPN-sensorimotor connectivity, visual-parietal and somatosensory differences, altered salience-default mode coupling, and lower global network efficiency. Our study supports a multilevel network model of PS and provide a more refined pathophysiological framework for future longitudinal studies, mechanistic investigations, and targeted neuromodulation strategies.

## Data Availability

The raw data supporting the conclusions of this article will be made available by the authors, without undue reservation.

## Footnotes

1
http://rfmri.org/dpabi

2
https://www.nitrc.org/projects/gretna
